# High content drug screening for Fanconi anemia therapeutics

**DOI:** 10.1186/s13023-020-01437-1

**Published:** 2020-06-30

**Authors:** Helena Montanuy, Cristina Camps-Fajol, Jordi Carreras-Puigvert, Maria Häggblad, Bo Lundgren, Miriam Aza-Carmona, Thomas Helleday, Jordi Minguillón, Jordi Surrallés

**Affiliations:** 1 Department of Genetics and Microbiology, Universitat Autònoma de Barcelona, Barcelona, Spain; 2 Join Research Unit on Genomic Medicine UAB-Sant Pau, Biomedical Research Institute, Hospital de la Santa Creu i Sant Pau, Barcelona, Spain; 3 Centro de Investigación Biomédica en Red de Enfermedades raras, Barcelona, Spain; 4 Division of Translational Medicine and Chemical Biology, Science for Life Laboratory, Department of Molecular Biochemistry and Biophysics, Karolinska Institutet, Stockholm, Sweden; 5 Currently at Division of Genome Biology, Science for Life Laboratory, Department of Molecular Biochemistry and Biophysics, Karolinska Institutet, Stockholm, Sweden; 6 Department of Biochemistry and Biophysics, SciLifelab, Stockholm University, Stockholm, SE Sweden; 7 Institute of Medical and Molecular Genetics and Skeletal dysplasia multidisciplinary Unit, Hospital Universitario La Paz, Universidad Autónoma de Madrid, IdiPaz, Madrid, Spain; 8 Centro de Investigación Biomédica en Red de Enfermedades Raras, Madrid, Spain; 9 Genetics Department, Hospital de la Santa Creu i Sant Pau, Barcelona, Spain

**Keywords:** Fanconi anemia, High content screening, Drug repositioning, Cell-based assay

## Abstract

**Background:**

Fanconi anemia is a rare disease clinically characterized by malformations, bone marrow failure and an increased risk of solid tumors and hematologic malignancies. The only therapies available are hematopoietic stem cell transplantation for bone marrow failure or leukemia, and surgical resection for solid tumors. Therefore, there is still an urgent need for new therapeutic options. With this aim, we developed a novel high-content cell-based screening assay to identify drugs with therapeutic potential in FA.

**Results:**

A TALEN-mediated FANCA-deficient U2OS cell line was stably transfected with YFP-FANCD2 fusion protein. These cells were unable to form fluorescent foci or to monoubiquitinate endogenous or exogenous FANCD2 upon DNA damage and were more sensitive to mitomycin C when compared to the parental wild type counterpart. FANCA correction by retroviral infection restored the cell line’s ability to form FANCD2 foci and ubiquitinate FANCD2. The feasibility of this cell-based system was interrogated in a high content screening of 3802 compounds, including a Prestwick library of 1200 FDA-approved drugs. The potential hits identified were then individually tested for their ability to rescue FANCD2 foci and monoubiquitination, and chromosomal stability in the absence of *FANCA*.

**Conclusions:**

While, unfortunately, none of the compounds tested were able to restore cellular FANCA-deficiency, our study shows the potential capacity to screen large compound libraries in the context of Fanconi anemia therapeutics in an optimized and cost-effective platform.

## Background

Fanconi anemia (FA) is a rare disease characterized by bone marrow failure (BMF), high incidence of cancer and congenital malformations. FA has a low incidence of 1/160,000 and an estimated carrier frequency of 1/200. Up to 80% of FA patients will develop BMF before the age of 10. At the second decade of life they will start to develop hematological malignancies, such as acute myeloid leukemia (AML) or myelodysplastic syndrome (MDS), with an accumulative incidence of up to 20% in the adulthood. Finally, from the third decade of life FA patients will develop solid tumors, especially head and neck squamous cell carcinoma with an accumulative incidence of more than 50% beyond their fourth decade of life [1].

There are currently 22 FA genes identified, forming a complex pathway that repairs stalled replication forks and inter-strand crosslinked lesions during the S-phase of the cell cycle. FANCA is the most frequently mutated gene accounting for up to 80% of all FA patients [1, 2]. The pathophysiological effects of the lack of a functional FA/BRCA pathway include hypersensitivity to DNA-damaging agents or aldehydes (from alcohol consumption or cell metabolism), oxidative stress or overexpression of proinflammatory cytokines, such as TNF-α [3–5]. These studies derived in the proposal of candidate drugs to prevent FA defects, and some of them are currently being tested in clinical trials, such as quercetin and metformin [6, 7].

Drug screenings are critical steps in the drug discovery strategies, and they can be divided in high throughput screenings (HTS), when they are based on specific mechanisms of action, or high content screenings (HCS), when the readouts are phenotypic changes. The preferred approach has long been HTS, as a molecular target is identified by basic research and further explored by research centers or the pharmaceutical industry, screening hundreds of thousands of molecules. Phenotypic approaches, however, require the identification of a specific cellular function that may be defective in a specific disease, of which the target is usually unknown, but has been very useful to find new drugs for rare diseases [8]. Compounds are usually screened in cells to correct an abnormal function, and these assays are optimized in technological facilities, such as microscopy or cell cytometry coupled with robotic arms, in order to allow for high-content screenings with libraries of thousands to tens of thousands of compounds.

In this study we present a cell-based system with a FANCA-defective background, in which we monitored the FA/BRCA pathway activity analyzing FANCD2 foci by fluorescence microscopy. We performed a high content screening in search for drugs that could rescue FANCA deficiency, and among the 3802 compounds tested we included FDA-approved drugs to check for drug repositioning candidates. We identify benefits and drawbacks of using this cell-based system for potential future screenings in the Fanconi anemia field.

## Results

### FA-cell based system to monitor FANCD2 foci formation

**Fig. 1:**
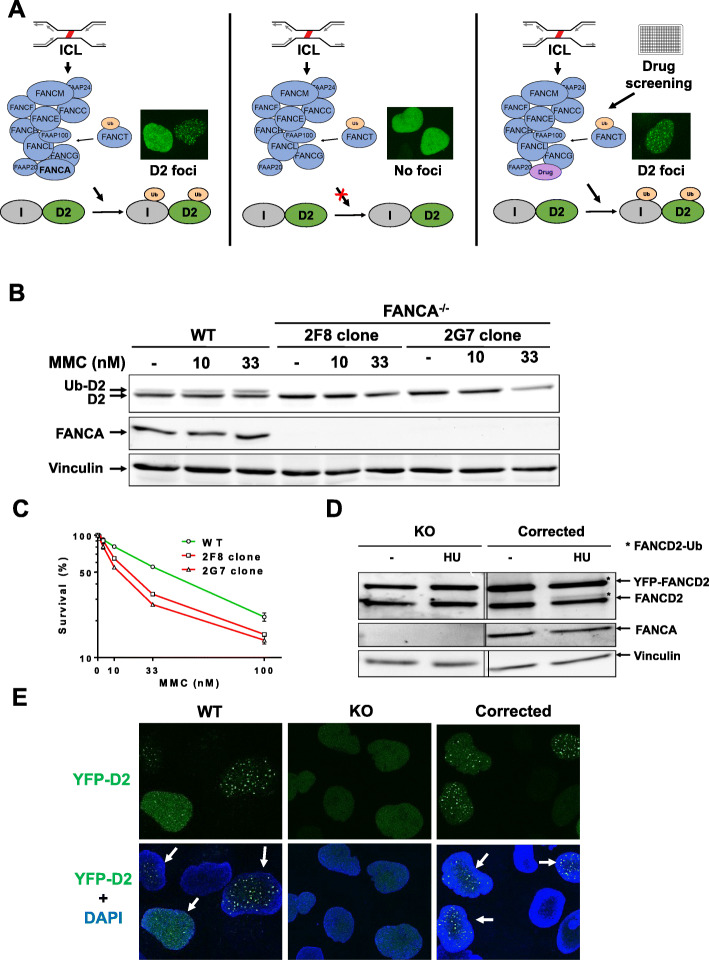
FA cell-based system setup. **a** Upon DNA damage causing ICLs on DNA, FA/BRCA pathway is activated, FANCD2 ubiquitinated, and fluorescent FANCD2 foci can be seen on the microscope (left). Deficient FA/BRCA pathway is not able to ubiquitinate FANCD2 and no FANCD2 foci can be detected (middle). A drug able to overcome FANCA deficiency on FA/BRCA pathway can be addressed with this cell-based system. FANCD2 foci can be clearly identified from the nucleus FANCD2 background signal (right). **b** Lack of FANCA expression of two positive clones after TALEN-mediated gene targeting. Cells were treated with MMC at 10 and 33 nM for 24 h, lyzed and FANCA expression and FANCD2 ubiquitination analyzed by Western blot. Vinculin was used as a loading control. **c** Survival assay of FANCA-deficient U2OS clones. 2F8 and 2G7 clones and WT cells were treated with a MMC dose curve from 1 to 100 nM. 72 h later cells were collected and counted. Graph shows mean survival percentage respect non-treated cells +/− SEM of at least three independent experiments with similar results. **d** 2F8 FANCA-deficient U2OS clone was stably transfected with YFP-FANCD2 (see materials and methods). FANCA-deficient cells (left lanes) and FANCA corrected cells (right lanes) were treated with 2 mM HU for 24 h, then lyzed and FANCA, endogenous FANCD2 and exogenous YFP-FANCD2 expression analyzed by Western blot. Vinculin was used as a loading control. **e** Fluorescence (green) of YFP-FANCD2 stably transfected on WT (left), FANCA-deficient (middle), and FANCA corrected (right) U2OS cell lines was analyzed by microscopy after treatment with 2 mM HU for 24 h. Bottom images show overlay of YFP-FANCD2 fluorescence with DAPI (nuclei staining)

Our objective was to create a cellular system based on fluorescence, to monitor the activation of the Fanconi anemia pathway (Fig. 1a). With this purpose, we developed a FANCA deficient cell line expressing fluorescently tagged FANCD2, as a central protein that requires functional upstream FA proteins (FANCA, FANCC and FANCG among them, whose mutations represent the majority of FA patients) to be activated by monoubiquitination. Covalent bonds between both DNA strands (interstrand-crosslinks, ICLs) in S-phase induce stalled replication forks and the activation of the E3 ubiquitin ligase FANCL [9]. FANCL then monoubiquitinates FANCD2 and FANCI, which will bind to the DNA lesion to recruit downstream proteins that will process the crosslink, and finally repair the DNA by homologous recombination. FANCD2 monoubiquitination can be easily seen by fluorescence microscopy. In the absence of DNA damage, FANCD2 is normally located in the nucleus as a diffuse staining, while monoubiquitinated FANCD2 is seen as intense spots, or foci, throughout the nucleus, representing stalled replication forks on chromatin during S-phase (Fig. 1a left) [2]. In a FANCA-deficient cell line, FANCL is not able to monoubiquitinate FANCD2 and FANCI. Thus, FANCD2 localization is seen in a nuclear diffuse pattern, even when cells are treated with ICL DNA damaging agents (Fig. 1a middle). A drug able to restore FANCL activity in the absence of FANCA would be able to again monoubiquitinate FANCD2/FANCI, restoring FANCD2 ability to form nuclei foci upon DNA damage induction (Fig. 1a right).

For our cell-based system we used the fast growing and genetically amendable U2OS cell line where we knocked-out FANCA gene expression by TALEN (Transcription Activator-like Effector Nuclease) gene editing [10]. FANCA-deficient clones were verified by sequencing, FANCA expression and FANCD2 ubiquitination (Fig. 1b and Figure Suppl. 1). Additionally, the two independent clones selected were more sensitive to mitomycin C treatment in survival assays than the wild type cell line (Fig. 1c). One of the clones was stably transfected with YFP-FANCD2 fluorescent fusion protein, in order to easily detect FANCD2 localization by fluorescence microscopy. The FANCA-deficient YFP-FANCD2 cell line expressed only the non-ubiquitinated form (Fig. 1d, left panel) and was unable to form fluorescent FANCD2 foci when cells were treated with hydroxyurea (HU, Fig. 1e, middle panel). When the cell line was infected with a FANCA-expressing retrovirus, endogenous FANCD2 and YFP-FANCD2 could be monoubiquitinated and FANCD2 foci could be seen again (Fig. 1d and e, right panels).

### High content screening

**Fig. 2:**
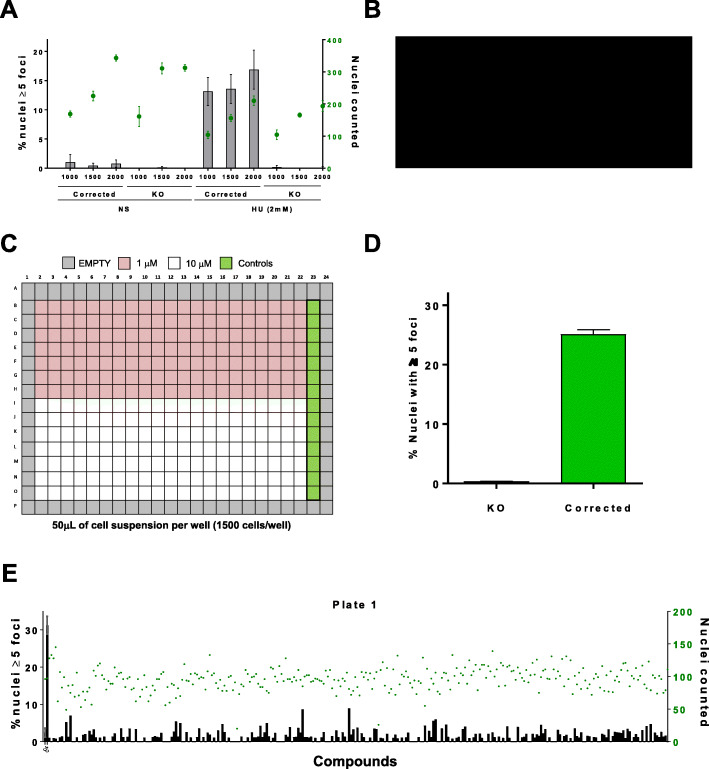
High content screening setup. **a** FANCA-deficient (KO) and corrected cells stably transfected with YFP-FANCD2 were plated at different concentrations, left untreated or treated 24 h with 2 mM HU. Fluorescent images were obtained on Image Express Microscopy and nuclear counts or nuclear foci detected with CellProfiler 2.0 software (see materials and methods). Grey bars (left legend) show percentage of cells with 5 or more nuclear foci. Green dots (right legend) show nuclei counted from 6 average images captured for each well. Graph shows mean +/− SD of three replicates. **b** Library collections used in the screening. **c** Drug layout on 384 well plates used in the screening. Violet wells, 1 μM drug treatment, white wells, 10 μM. Green wells, positive controls (top six wells, FANCA-deficient cell line, bottom six wells, FANCA-corrected cell line). Grey wells that come from the plate edges where left empty to avoid sample evaporation. **d** Percentage of nuclei with 5 or more foci from control samples on each plate. **e** Representative graph from a 384 well plate with percentage of cells with foci (black bars, left legend) analyzed with CellProfiler 2.0 software, in parallel with nuclear count (green dots, right legend) to show potential drug toxicity

After testing different conditions (Fig. 2a and data not shown), we seeded 1500 cells per well in 384 well-plates where we previously dispensed the drug collection. After 24 h cells were treated with hydroxyurea, and 24 h later plates were fixed, washed, images collected and processed. From the collected images around 100–200 nuclei per well were counted with CellProfiler 2.0 software (six images/well taken with the 40x objective). 384 well plate layout allowed to test 147 compounds/plate (two plates for replicates), with two concentrations per plate (1 and 10 μM), totaling 52 plates for the 3802 drugs from 4 different libraries (Fig. 2b and c). Figure 2e shows a representative plate with the percentage of nuclei with 5 foci or more (black bars, left panel), along with the number of nuclei counted (green dots, right panel). Negative and positive controls (FANCA-deficient and corrected cells, respectively) displayed a good signal respect the background (Fig. 2d), and a Z’ factor of 0.4 (see materials and methods). Although for non-cell based, high throughput assays, a Z’ factor above 0.5 is recommended, cell-based assays are intrinsically more complex in their read-out, and in these cases Z’ factor values of 0.4 still provide a good signal window [11, 12].

### Hit validation

**Fig. 3:**
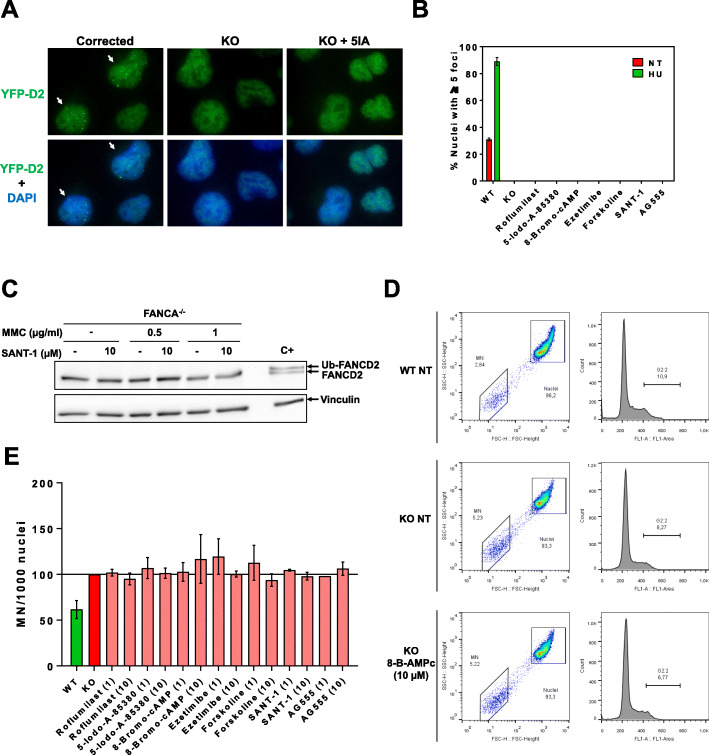
Hit validation. **a** FANCD2 foci detection from YFP-FANCD2 fluorescence from corrected (left lanes), FANCA-deficient (KO, middle lanes), and FANCA-deficient U2OS cell line, incubated with one of the hits (right lanes). Cells were treated with selected hits at 10 μM, 4–6 h later treated with 2 mM HU, and left 16–24 h. Cells were then fixed and immunostained with FANCD2 antibody (see materials and methods). Upper images show FANCD2 localization and bottom images show overlay of FANCD2 with DAPI. White arrows on left lanes show cells with FANCD2 foci. **b** Graph quantification of cells with 5 or more foci, from the experiment performed in **a** (and data not shown). Results show mean percentage of cells with foci +/− SEM from three independent experiments with similar results. **c** FANCA-deficient U2OS cells were treated with SANT-1 at 10 μM, and or MMC, for 24 h. Cells were lyzed and FANCD2 monoubiqutination analyzed by Western blot. Vinculin was used as a loading control. Similar results were obtained from the other hits testes (data not shown). **d** Spontaneous chromosomal fragility analysis in FANCA-deficient lymphoblastoid cell lines. Graphs show gates from cell cytometry experiments for micronuclei production (left) and G/M cell cycle arrest (right) in Wild type (WT, upper), FANCA-deficient (KO, middle) or FANCA-deficient lymphoblastoid cells stimulated with 10 μM 8-Bromo-AMPc (bottom). **e** Graph showing mean +/− SEM spontaneous micronuclei production after stimulation of selected hits (at 1 or 10 μM), from at least three independent experiments as in **d**

Once we performed the screening, collected the images and processed them, we selected compounds that induced at least 5% nuclei with 5 or more FANCD2 foci, and maintained cell number with respect the control (indicating no or low toxicity). Unfortunately, all the positive candidates once manually revised were false positives, as all the compounds displayed high autofluorescence or different FANCD2 distribution, not compatible with nuclear foci (data not shown). These results indicated that none of the drugs tested were able to recover FANCD2 foci in our FANCA-deficient cell-based system. We then searched for drugs that could have a lower but detectable activity, by filtering out which drugs could induce between 2 to 5% of nuclei with 5 or more FANCD2 foci. We selected 6 potential compounds that could induce FANCD2 foci in a small percentage of the FANCA-deficient cells: roflumilast, a phosphodiesterase-4 (PDE-4) inhibitor; 5-iodo-A-85380, an agonist for the α_4_β_2_ and α_6_β_2_ nicotinic acetylcholine receptors; ezetimibe, a lipid-lowering compound; forskolin, a labdane diterpene used in research to increase cyclic AMP (cAMP); SANT-1, a sonic hedgehog signaling inhibitor; and AG555, an EGFR kinase inhibitor. In the follow-up studies we additionally used 8-Bromo-cAMP, a brominated derivative of cAMP, that has a similar effect as forskolin [13]. Unexpectedly, we were unable to validate FANCD2 foci formation by these drugs in the same cell line used in the screening (Fig. 3a, b and data not shown). We also checked for FANCD2 monoubiquitination by Western blot, and as seen in Fig. 3c (and data not shown), neither of the candidates were able to rescue the lack of FANCD2 ubiquitination. Finally, we wanted to see if these molecules were able to reduce genome instability in a FA-deficient cell line. FA cells accumulate genomic instability, both spontaneously and induced by DNA-damaging agents such as hydroxyurea, mitomycin C or diepoxybutane. This genomic instability is used in diagnostics in chromosome fragility tests [14], and it has also been reported in micronucleus production and single-cell gel electrophoresis (comet assay) studies [15, 16]. We adapted the micronucleus assay to cell cytometry (Figure Suppl. 2) [17], where we could count thousands of events in a short period of time, having more sensitivity to detect mild changes. As seen in Fig. 3d and e, in basal conditions FANCA-deficient lymphoblastoid cells had close to two time more micronucleus than wild type cells. When treated with candidate hits, the micronucleus levels in these cells were not significantly reduced. However, some drugs did have a mild reduction effect, such as roflumilast, forskolin or SANT-1, all three at 10 μM concentration (Fig. 3e).

## Discussion

So far, drug screenings that target the FA/BRCA pathway published in the literature have been focused in searching for inhibitors [18, 19]. Instead, our cell-based system was focused in the FA/BRCA-pathway recovery, in a FANCA-deficient background that yields FANCL ubiquitin ligase inactive, so FANCD2 cannot be monoubiquitinated nor localize to the nucleus in stalled replication forks. Thus, by looking for FANCD2 foci formation, to our knowledge, this is the first report to screen for drugs with the aim to recover FA/BRCA pathway activity in FA-deficient cells.

It is extremely difficult to conduct a complete drug discovery process, which normally takes 15 years or more, with highly elevated costs [20]. As the potential economic return would be very limited in a rare disease, a drug repositioning strategy has been explored in some cases, such as the recent identification of the antifungal ciclopirox to treat congenital erythropoietic porphyria [21]. In the FA field itself, repositioning approaches for BMF or DNA-damage prevention have actually been described, and positive results have led to clinical trials such as with quercetin (NCT01720147 and NCT03476330, www.clinicaltrials.gov) [6] and metformin (NCT03398824) [7]. In our screening of 3802 compounds we included 1200 FDA-approved drugs (Prestwick library). However, we were not able to find good candidates which recovered FANCD2 foci induction in our FA-deficient cell line. Thus, our results discard a therapeutic potential of FDA approved drugs to treat FA, in terms of upstream FA/BRCA-pathway activity. Additionally, around 5% of the drugs led to false positive hits, as some had a high autofluorescence, and others changed FANCD2 distribution, not similar to foci spots. An example was merbromin, which emits strong autofluorescence (data not shown). These false positives could be a drawback of the system, taking into account that all molecules with green autofluorescence will be detected as positive hits. Fortunately, false positives in our assay were quickly filtered-out by visual inspection, as they did not fit the typical nuclei foci pattern. However, potential molecules with real activity but autofluorescent would be discarded, a true limitation of our cell-based system.The 6 candidates with apparently mild activity detected in the HCS could not be later confirmed in validation studies, including FANCD2 foci formation by immunohistochemistry, chromosome fragility and FANCD2 monoubiquitination. Nevertheless, we cannot rule out the possibility of a FANCL-independent FANCD2 activity, as it has recently been reported that FANCD2 chromatin recruiting is partially independent of monoubiquitination [22]. Interestingly, we did see an effect on micronuclei reduction in some candidates (roflumilast and forskolin, Fig. 3e), which would suggest these drugs may reduce chromosome instability, although this effect was mild. The potential applicability of these results requires further validation and investigation.

Our cell-based system was designed with the objective to correct FA/BRCA pathway activity in the absence of FANCA protein. However, there is the possibility that drug candidates could impact positively in the cellular phenotype, more than in the pathway itself, in FA-deficient cells. As an example, micronucleus formation, increased in buccal mucosa of FA-patients and blood cells in murine models [23, 24], is a general biomarker of chromosome instability, and drugs able to reduce this induction could be good therapeutic candidates. Another example could be monitoring other phenotypic changes seen in FANCA-deficient cells, such as mitochondrial activity [25].

## Conclusions

We developed and validated a novel FA/BRCA-pathway recovery cell-based assay to screen thousands of compounds in a HCS approach. Our results suggest that at the conditions tested, none of the FDA-approved drugs can rescue FA/BRCA pathway activity in the absence of FANCA. The same is true for other thousands of compounds known to reflect chemical diversity. However, even that we did not observe FANCD2 foci recovery, other positive effects such as in reduction of genomic instability, oxidative stress or apoptosis cannot be discarded. That would be the case of some drugs, such as quercetin and metformin, recently described in the literature and currently in clinical trials, or NAC, dasatinib and TGF-β inhibitors, also reported earlier [26–28].

## Methods

### Reagents

Ethidium Monoazide Bromide (EMA, ref. e1374) Opti-MEM (ref. 31,985–070), BCA Protein Quantification Assay (ref. 23,227), SYTOX® Green (ref. S7020) and Hoechst® (ref. H1399) were from ThermoFisher Scientific. IGEPAL (ref. I8896), DAPI (ref. D9542), diepoxybutane (ref. 202533–16), hydroxyurea (ref. H8627–56), mitomycin C (ref. M-0503), β-mercaptoethanol (ref. M3148), triton X-100 (ref. T8787), BSA (ref. A7906), Complete Mini EDTA-free Protease Inhibitor Cocktail (ref. 04693159001), PhosSTOP Phosphatase Inhibitor Cocktail Tablets (ref. 04906837001), roflumilast (SML1099), 5-iodo-A-85380 (SML0023) and 8-bromo-cAMP (B7880) were from Sigma-Aldrich. Ezetimibe (HY-17376) and forskolin (HY-15371) were from Medchem Express. RNase A (ref. 12,091,021), Lipofectamine® 2000 (ref. 11,668,019) and glycerol (ref. 15,514–011) were from Invitrogen. RPMI (ref. L04495) and DMEM (ref. L0104) mediums, non-essential amino acids (ref. X0557), fetal bovine serum (FBS, ref. S181B), sodium pyruvate (ref. L0642) and penicillin-streptomycin antibiotic (ref. L0022) were from Biowest. Plasmocin (ref. ant-mpt) was from Invivogen. RIPA 10X lysis buffer (ref. 20–188) was from Millipore. Benzonase nuclease (ref. 70,746) was from Novagen. Antibodies rabbit anti-FANCD2 (ab2187) and mouse anti-Vinculin (ab18058) were from Abcam. Rabbit anti-FANCA (F3165) was from sigma. SANT-1 (sc-203,253), AG555 (sc203500), Goat anti-rabbit IgG-HRP (sc-2004) and goat anti-mouse IgG-HRP (sc-2005) secondary antibodies were from Santa Cruz. Goat anti-rabbit Alexa-Fluor488 was from ThermoFischer. Paraformaldehyde (ref. SC281692) was from Santa Cruz, vectashield antifade vector (ref. H-1000) was from Vector Laboratories.

### Cell lines

WT and FANCA-deficient lymphoblastoid cell lines were cultured in RPMI medium with 20% FBS heat inactivated, penicillin-streptomycin, sodium pyruvate, nonessential amino acids and β-Mercaptoethanol. U2OS cell lines (WT, U2OS-FANCAKO-YFP-FANCD2 and U2OS-FANCAKO-YFP-FANCD2-FANCAcorrected) were cultured in DMEM medium with 10% FBS heat inactivated with plasmocin antibiotic. All cell lines were cultured at 37 °C and 5% of CO_2_. FANCA gene was knocked-out in U2OS cell line by TALEN gene editing (Transposagen Biopharmaceuticals), a more specific alternative than CRISPR/CAS9 system, with fewer off-targets [10]. We used surrogate reporters for clone selection optimization, as provided by the manufacturer and described elsewhere [29]. Briefly, 3 × 10^5^ cells where seeded on 6 well plates. TALEN plasmids where designed to target EXON1 of FANCA gene, being the target sequence 5′ TAGGCGCCAAGGCCATGT ccgactcgtgggtccc GAACTCCGCCTCGGGCCA 3′ (uppercase letters show forward and reverse binding regions and the lowercase letters the cutting region, see Supplementary Figure 1). Cells were transfected with surrogate-FANCA and forward/reverse TALEN-FANCA plasmids. Once green fluorescence was detected cells were sorted in a FACSJazz cell sorter (Becton Dickinson) and individual cells seeded in 96 well plates. Grown clones were then tested by Western blot for FANCA expression, and positive clones with no FANCA were sequenced to identify mutations/deletions. For fluorescent FANCD2 stable transfection, 5 × 10^5^ U2OS WT or FANCA-deficient cells were seeded on 6 well plates and transfected with pEAK8-YFP-FANCD2 plasmid [30] with lipofectamin reagent (Invitrogen), following manufacturer’s instructions. Fluorescent cells were sorted, and selection was repeated two more times to maintain stable fluorescence. To correct FANCA-deficient YFP-FANCD2 U2OS cells, a FANCA expressing retroviral plasmid was infected in these cells, and growth sensitivity checked by 10 nM MMC treatment for 3–5 days.

### Survival assay

For survival assays 1 × 10^5^ cells were seeded in 6 well plates in duplicates, 24 h later were treated with a MMC dose curve from 1 to 100 nM and left in culture for 72 h. Cells were then trypsinized and counted in a Z series Coulter Counter (Beckman-Coulter).

### Western blot

2 × 10^5^ cells were seeded on 6 well plates, 24 h later were treated with hydroxyurea, MMC or left untreated, and 24 h later cells were lyzed and SDS-PAGE and blotting with indicated antibodies performed as previously described [31].

### FANCD2 foci

2 × 10^5^ cells from YFP-FANCD2 stably transfected WT, FANCA-deficient or corrected U2OS cell lines were plated in coverslips in 6 well plates. After 4–6 h cells were treated with selected drugs or left untreated for 24 h. Cells were then treated with 2 mM hydroxyurea and 24 h later were washed, fixed with 4% PFA in PBS for 15 min and permeabilized with 0.5% TX-100 solution in PBS for 10 min, DAPI stained and foci imaged with fluorescence microscopy (Leica Microsystems). For immunofluorescence, YFP-FANCD2 stably transfected FANCA-deficient or corrected cell lines were plated in coverslips in 6 well plates. After the experiment, fixed and permeabilized cells were blocked for 15 min with 5% BSA and 0.05% Tween-20 in PBS (blocking solution). Primary antibody was incubated in blocking solution overnight at 4 °C in humidity. After washing, samples were incubated with secondary antibody in blocking solution for 20 min at 37 °C. Samples were then washed twice with blocking solution and deionized water, DAPI stained and foci imaged by fluorescence microscopy.

### High content screening

High content screening of 3802 compounds was conducted at Science for Life Laboratory, (Karolinska Institutet, Stockholm, Sweden). Libraries used (chemical properties in Supplementary Figure 2) were: Prestwick library of FDA approved drugs (1200 compounds); Selleck library, including FDA approved and 378 kinase inhibitors (1368 compounds); Tocris library (1119 compounds) and compounds from Thomas Helleday lab (115 compounds). These molecules were dissolved in DMSO at 10 mM concentration, and dispensed to 384 well plates with Echo® 550 Liquid Handler (Labcyte) at two different final concentrations (1 and 10 μM, see Fig. 2c layout), sealed and stored at 4 °C. Next day, cells were seeded in 50 μl of medium using Multidrop™ Combi dispenser (ThermoFisher Scientific) and left in the incubator. The following day cells were treated with 2 mM hydroxyurea for 24 h. Cells were then fixed with 4% PFA in PBS, stained with 2 *μ* M Hoechst and stored in darkness at 4 °C in PBS with 0.05% sodium azide.

Images were taken with ImageXpress Micro XLS microscope (Molecular Devices) through a VALet™ robotic arm (ThermoFisher Scientific). Six images/well were taken for nuclei and FANCD2 foci counting, with a 40x objective. For FANCD2 foci detection and nuclei counting, a specific pipeline was prepared with CellProfiler 2.0 software (Broad Institute). Using proper positive and negative controls, image analysis gave a Z’ factor of 0.4. Z’ factor reflects the separation magnitude between the positive and the negative controls. After the initial hit identification, positive hits were manually checked to discard auto fluorescent compounds.

### Micronucleus analysis by cell cytometry

Chromosomal fragility and G2/M cell cycle analysis were carried out using WT and FANCA-deficient lymphoblastoid cell lines and was previously described [17]. 6 × 10^4^ cells were seeded in triplicates in 96 well plates, and 4–6 h after were treated with candidate hits at 1 or 10 μM. 24 h later cells were treated with 0.1 μg/ml DEB. 72 h later cells were harvested and counted in order to check for proper proliferation. Cells were then incubated with 25 μg/ml EMA (in PBS + 2% FBS) to stain necrotic and late-stage apoptotic cells. After 20 min of photoactivation at 4 °C, EMA excess was removed by adding PBS-2% FBS. After EMA labelling, cells were exposed to lysis solution 1 (0.3 *μ* l/ml IGEPAL, 0,582 mg/ml NaCl, 1 mg/ml RNAse A, 1 mg/ml sodium citrate, and 0.2 μM SYTOX Green). After one hour at room temperature, cells were diluted with lysis solution 2 (85.6 mg/ml sucrose, 15 mg/ml citric acid, 0.2 μM SYTOX Green). After 30 min at room temperature, samples were stored at 4 °C until its analysis 24 h later in a BD FACSCalibur flow cytometer. 20,000 live event cells were acquired on the basis of DNA content. Several gates were needed in order to separate micronuclei from apoptotic chromatin and debris (see Supplementary Figure 3).

## Supplementary information

**Additional file 1: Supplementary Figure 1.** FANCA exon 1 sequence from U2OS clones 2F8 (A) and 2G7 (B). Forward and reverse primers are shown in blue and yellow, respectively. Bold letters show first codon of the FANCA open reading frame in the exon (ATG). Violet filled boxes show targeted TALEN sequence. Open black boxes show deleted regions of 2F8 (A, 7 bp) and 2G7 (B, 83 bp) clones.

**Additional file 2: Supplementary Figure 2.** Library chemical properties. Average chemical properties from the 4 libraries used: A and B, AlogP (partition coefficient, measures the hydrophobicity/lipophilicity); C, HBA (number of hydrogen bond acceptors); D, HBD (number of hydrogen bond donors); E, Heavy Atom Count (number of non-hydrogen atoms); F, MW (molecular weight); G, RB (number of rotatable bonds).

**Additional file 3: Supplementary Figure 3.** Cell cytometry gate analysis for micronuclei assay in FANCA-deficient LFA55 lymphoblastoid cell line. A) Forward scattering (FSC) versus side scattering (SSC). A gate is created to include events with 1/100 nuclei size (FSC) and 1/100 nuclei granularity (SSC). B) SYTOX histogram. In this gate events with less fluorescence than 1/100 of the G1 population are discarded. C) SYTOX gate to plot width versus area to discard doublets and select single events. D) SYTOX vs SSC gate. Nuclei and events with a granularity and fluorescence 1/100 of the G1 are selected. E) SYTOX vs FSC. Nuclei and events with a size and fluorescence 1/100 of the G1 are selected. F) EMA vs DAPI. By discarding EMA positive events, data from apoptotic or necrotic cells is removed. G) FSC vs SSC. After all gate selection, FSC vs SSC dot plot show a clear distinction between nuclei (upper right box) and micronuclei (MN, lower left box). MN are defined as events between 1/100 to 1/10 fluorescence from that of 2 N nuclei. H) SYTOX histogram. Nuclei from G can be further analyzed to see cell cycle status (selected region shows 1/2 G2/M cells).

## Data Availability

The datasets used and/or analyzed during the current study are available from the corresponding author on reasonable request.
