# Supplementary material for: High content drug screening for Fanconi anemia therapeutics
Source: Orphanet J Rare Dis. 2020 Jun 30;15:170. doi: 10.1186/s13023-020-01437-1 (PMC7325660; doi:10.1186/s13023-020-01437-1)
Supplement: Supplementary file 1 [file 13023_2020_1437_MOESM1_ESM.docx]

Supplementary Figure 1

A

2F8 clone

```
tcattcccggcaggaaccacgcttgaggggcggcagtctgcccgcgcgagacgcccccgcggactacaccgcggcggcaaagccaaacgcaaaaact
acctcaccgcgcgcaggcgcctcccccaggaccaacatggccacgacgcaaggcctcgacctgaggggcgtggcctggccgccgccagccaacgggt
gtgcgcgcctggccgcagccaataggaaggcagcgcgggctcgggcgcagggAGCCGCCGCCGGGGCTGTAGGCGCCAAGGCCATGTCCGACTCGTG
GGTCCCGAACTCCGCCTCGGCCCAGGACCCAGGGGGCCGCCGGAGGGCCTGGGCCGAGCTGCTGGgtaggtgggcgcggcaggccgcgggagtgggc
ggcgtccggcccgggacggtttcgccggttccccgatcccttcccgccagagcctccgccggtcggatccccggacgccgcgcccgggggggctgtgc
ggggtgggcgcccggctggggcggcgcggctgcctcggacccggcccctcctgcgcctgggcggacgcccccagccgccgcccgcggggcgctccct
tctttcccgaacgccgcccccgccggccgccctgtcaggcgggcctggggtgcgcggcctggggctcccctcagcgcagaggccgcccctcgccagc
cgtccccgggctcccctgcctcgggccctcctgggccgtcttccccggcgtccgcggtggggccgtctccgttagtttcccgagacctgcgccctgg
ggaggagccccggcccctcttcgggagggtgtcgctggtgggtttctcc
```

B

2G7 clone

```
tcattcccggcaggaaccacgcttgaggggcggcagtctgcccgcgcgagacgcccccgcggactacaccgcggcggcaaagccaaacgcaaaaact
acctcaccgcgcgcaggcgcctcccccaggaccaacatggccacgacgcaaggcctcgacctgaggggcgtggcctggccgccgccagccaacgggt
gtgcgcgcctggccgcagccaataggaaggcagcgcgggctcgggcgcagggAGCCGCCGCCGGGGCTGTAGGCGCCAAGGCCATGTCCGACTCGTG
GGTCCCGAACTCCGCCTCGGCCCAGGACCCAGGGGGCCGCCGGAGGGCCTGGGCCGAGCTGCTGGgtaggtgggcgcggcaggccgcgggagtgggc
ggcgtccggcccgggacggtttcgccggttccccgatcccttcccgccagagcctccgccggtcggatccccggacgccgcgcccgggggggctgtgc
ggggtgggcgcccggctggggcggcgcggctgcctcggacccggcccctcctgcgcctgggcggacgcccccagccgccgcccgcggggcgctccct
tctttcccgaacgccgcccccgccggccgccctgtcaggcgggcctggggtgcgcggcctggggctcccctcagcgcagaggccgcccctcgccagc
cgtccccgggctcccctgcctcgggccctcctgggccgtcttccccggcgtccgcggtggggccgtctccgttagtttcccgagacctgcgccctgg
ggaggagccccggcccctcttcgggagggtgtcgctggtgggtttctcc
```
