# Supplementary material for: High content drug screening for Fanconi anemia therapeutics
Source: Orphanet J Rare Dis. 2020 Jun 30;15:170. doi: 10.1186/s13023-020-01437-1 (PMC7325660; doi:10.1186/s13023-020-01437-1)

Supplementary Figure 3

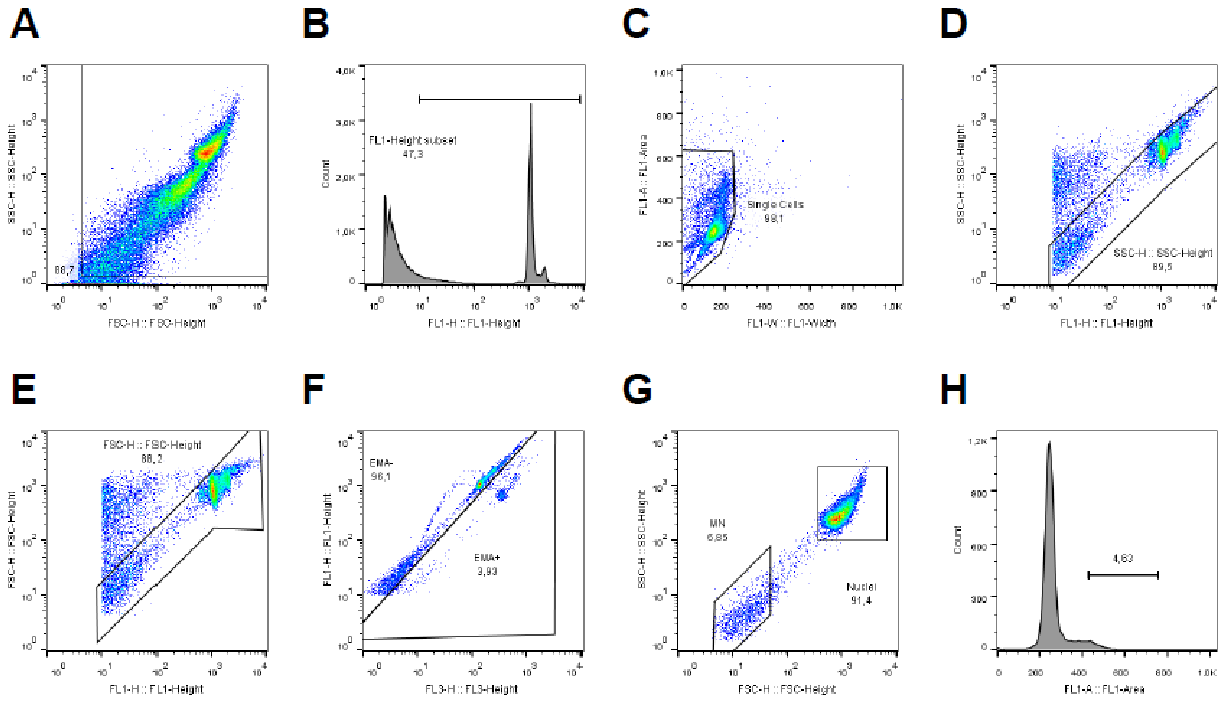

Supplement: Supplementary file 3 [file 13023_2020_1437_MOESM3_ESM.docx]
